# Dynamic Boolean modeling of molecular and cellular interactions in psoriasis predicts drug target candidates

**DOI:** 10.1016/j.isci.2024.108859

**Published:** 2024-01-11

**Authors:** Eirini Tsirvouli, Vincent Noël, Åsmund Flobak, Laurence Calzone, Martin Kuiper

**Affiliations:** 1 Department of Biology, Norwegian University of Science and Technology, 7034 Trondheim, Norway; 2 Department of Clinical and Molecular Medicine, Norwegian University of Science and Technology, 7030 Trondheim, Norway; 3 Institut Curie, Université PSL, 75005 Paris, France; 4 INSERM, U900, 75005 Paris, France; 5 Mines ParisTech, Université PSL, 75005 Paris, France; 6 The Cancer Clinic, St Olav’s University Hospital, 7030 Trondheim, Norway; 7 Department of Biotechnology and Nanomedicine, SINTEF Industry, 7034 Trondheim, Norway

**Keywords:** Medicine, Disease, Drugs

## Abstract

Psoriasis arises from complex interactions between keratinocytes and immune cells, leading to uncontrolled inflammation, immune hyperactivation, and a perturbed keratinocyte life cycle. Despite the availability of drugs for psoriasis management, the disease remains incurable. Treatment response variability calls for new tools and approaches to comprehend the mechanisms underlying disease development. We present a Boolean multiscale population model that captures the dynamics of cell-specific phenotypes in psoriasis, integrating discrete logical formalism and population dynamics simulations. Through simulations and network analysis, the model predictions suggest that targeting neutrophil activation in conjunction with inhibition of either prostaglandin E2 (PGE2) or STAT3 shows promise comparable to interleukin-17 (IL-17) inhibition, one of the most effective treatment options for moderate and severe cases. Our findings underscore the significance of considering complex intercellular interactions and intracellular signaling in psoriasis and highlight the importance of computational approaches in unraveling complex biological systems for drug target identification.

## Highlights

• The PsoriaSys model captures the multiscale dysregulation of normal skin homeostasis
• Population dynamics of keratinocytes and immune cells are correctly predicted
• Dynamic Boolean modeling is used to predict new psoriatic drug targets
• Simulations suggest inhibiting neutrophils with PGE2 or with STAT3 as a new therapy

## Graphical abstract

**Figure:**
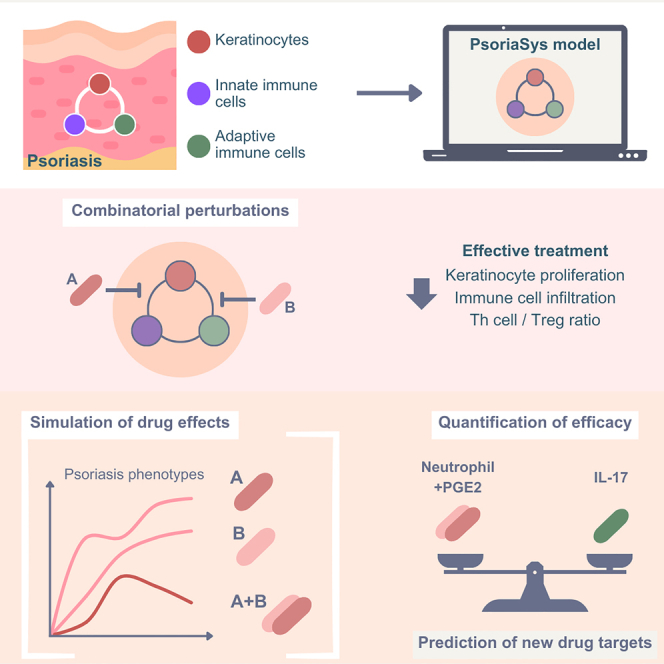

## Introduction

Psoriasis is a chronic skin disease, characterized by a dysregulation of inflammation and immune processes and estimated to affect 2–3% of the global population. The full etiology of psoriasis is multifactorial, with genetic predisposition and environmental factors modulating disease susceptibility, therapy response, and disease course.^1^ Psoriasis can be described as a complex, dysregulated multicellular system in which the interaction between immune cells and keratinocytes results in the emergence of the disease’s various phenotypes. The main hallmarks of psoriasis include immune activation and keratinocyte hyperproliferation, which involve the activation of inflammatory cascades and the release of cytokines, chemokines, and growth factors.^2^ These factors create a perpetual loop that sustains and exacerbates immune responses while promoting epidermal hyperproliferation. Due to the complex nature of psoriasis, a multiscale systems approach is expected to aid understanding of its pathophysiology and therapeutic options.

Psoriasis evolves in two main stages: the initiation stage and the maintenance stage. Both stages are known to involve multiple positive feedback loops of signaling between keratinocytes and immune cells. Initially, keratinocytes can respond to a variety of inflammatory triggers that can be either external (e.g., skin injury or drugs) or internal (e.g., infections or mental stress).^1^ In response to such triggers, keratinocytes release pro-inflammatory mediators that promote immune cell activation and infiltration into the skin.^3^ Such mediators include the antimicrobial peptide LL-37 that, in complex with either DNA or RNA, recruits and activates plasmacytoid dendritic cells (pDCs). Active pDCs produce high levels of interferon (IFN)-α that activate myeloid DCs (mDCs). Mature mDCs shape the differentiation landscape of naive T-helper (Th) cells toward the Th1, Th17, and Th22 subtypes.^4^^,^^5^^,^^6^ These subtypes and their secreted cytokines dominate the cytokine microenvironment, and keratinocytes are considered the main responders to Th cell-derived cytokines.^7^ More specifically, IFN-γ and tumor necrosis factor alpha (TNFα) are mainly produced by Th1 cells, whereas interleukin-17 (IL-17) is produced by Th17 cells, and IL-22 is produced by both Th17 and Th22 subtypes.^7^ Keratinocytes stimulated by these cytokines have a disrupted life cycle characterized by unabated proliferation, aberrant differentiation, and reduced apoptosis. Additionally, psoriatic keratinocytes adopt an inflammatory phenotype and secrete to their environment additional cytokines and chemokines, which promote the survival and proliferation of immune cells and further recruit additional cell types, such as neutrophils, thereby sustaining chronic inflammation.^4^ The interplay between keratinocytes and immune cells creates a system where reciprocal cause and effect sustains the psoriatic disease phenotype.

Despite the significant advancements made in identifying the causes of psoriasis and developing effective treatments that work for many patients, there are still challenges to overcome. The high variability in treatment responses among individual patients and the ongoing need for affordable medication due to the chronic nature of the disease demand the design of new therapies to effectively manage psoriasis. Current treatment options for psoriasis include topical creams, phototherapy, systemic medications, and biologic agents. Biologics are a relatively new class of drugs that target specific molecules in the immune system that play a key role in the development and progression of psoriasis, and these have revolutionized the treatment of psoriasis. Examples include TNFα inhibitors (e.g., etanercept, adalimumab), IL-17 inhibitors (e.g., secukinumab, ixekizumab), and IL-23 inhibitors (e.g., guselkumab, tildrakizumab). While these treatments can effectively reduce symptoms and improve the quality of life for many patients, they often come with significant side effects, and not all patients respond equally to the same treatment.^8^ The high variance in treatment response highlights the need for personalized medicine approaches to tailor treatment options to individual patients based on their unique genetic, molecular, and clinical characteristics. Drug switching and combination treatment have been proposed as potential solutions for these patients.^9^^,^^10^

Previously, we have modeled the effects of IL-17 signaling on PGE2 production in psoriatic keratinocytes and have worked on an extended version of this model to capture heterogeneous manifestations of the disease and differential responses to treatments when coupled with patient gene expression data (Preprint at^11^). Here, we present a multiscale population model that allows us to follow the dynamics of cell-specific phenotypes in a time-resolved manner. The model represents the key intercellular interactions and intracellular signaling cascades involved in the disease and is based on a discrete logical formalism and population dynamics simulated with UPMaBoSS.^12^^,^^13^ Using network analysis through propagation metrics, we identified pairwise interventions that could serve as alternative treatment options, which might be experimentally tested. Among those combinations, the inhibition of neutrophils together with PGE2 production or STAT3 was predicted to have an effect similar to IL-17 inhibition.

## Results

### The PsoriaSys model - A multiscale model of psoriasis

**Figure 1:**
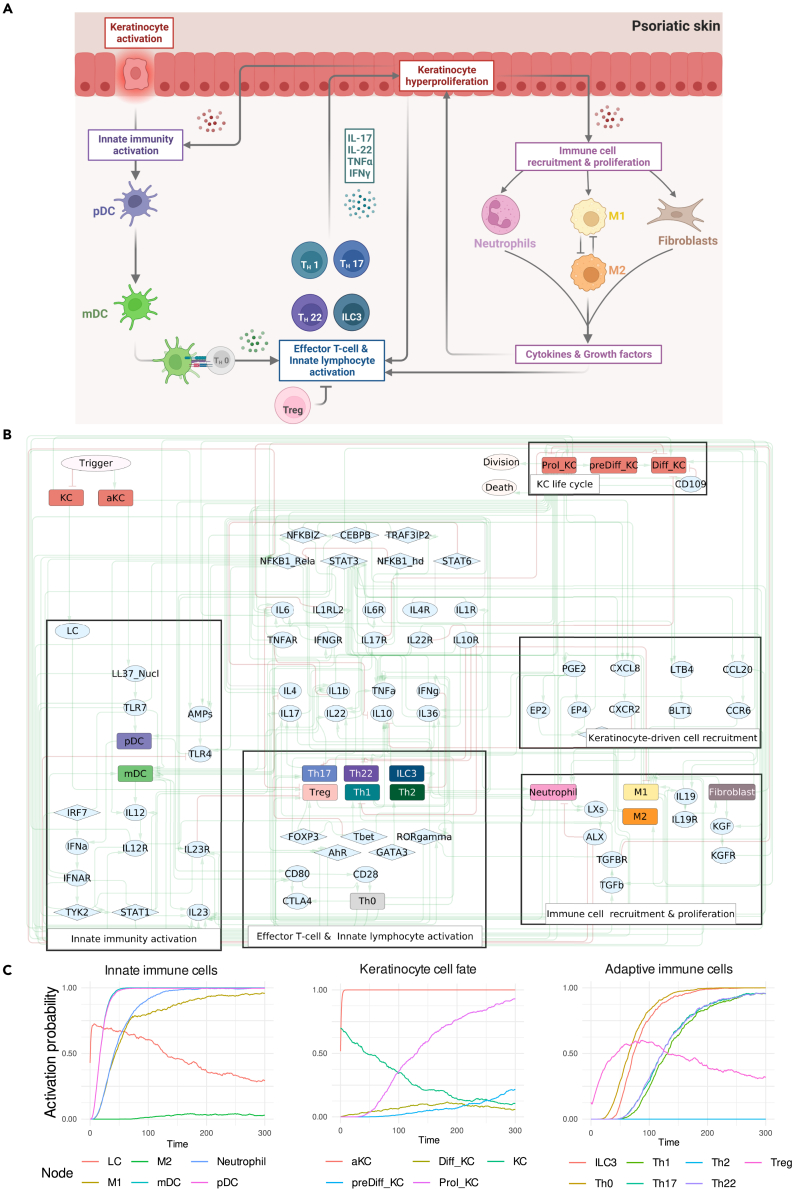
Main psoriasis players and their interactions represented in the PsoriaSys model (A) Schematic representation of the main cell types and interactions represented in the psoriasis model. pDC = plasmacytoid dendritic cells, mDC = myeloid dendritic cells, IL = Interleukin, Th = Thelper cells, Treg = T regulatory cells. (B) The PsoriaSys model. Nodes represent cell types or molecular entities. The nodes are colored based on the type of entity they represent (*see* figure legend). Green edges represent activating interactions and red edges represent inhibiting interactions. The diamond node represents the trigger of the disease. (C) The sequence of events in response to the psoriatic trigger. At Time = 0 a psoriatic trigger is applied. Abbreviations: LC = Langerhans cell, M1 = M1 macrophage, M2 = M2 macrophage, pDC = plasmacytoid dendritic cell, mDC = myeloid dendritic cell, KC = normal keratinocyte, aKC = trigger-activated keratinocyte, Prol_KC = proliferating keratinocyte, preDiff_KC = aberrantly differentiated keratinocyte, Diff_KC = terminally differentiated keratinocyte, ILC3 = Type 3 innate lymphoid cell, Th0 = naive T helper cell, Th = T helper cell, Treg = regulatory T cell.

The PsoriaSys model represents the main cell types and signaling components that have a well-described role in the development of the disease. A high-level overview of the known disease mechanisms represented by the model is provided in Figure 1A. The model consists of 87 nodes and 235 edges (Figure 1B). The model’s nodes can represent a cell type (e.g., neutrophils), a cell in a specific state (e.g., proliferating keratinocytes), a ligand (e.g., IL-17) or its receptors (e.g., IL-17R), a transcription factor (e.g., STAT3), or other intermediate signaling proteins. The model encompasses 18 cell nodes, and it describes the main intercellular interactions and intracellular signaling during the various psoriasis stages. The selection criteria for these nodes were based on an extensive literature review focusing on cell types and signaling entities implicated in the disease. Priority was given to cell types and signaling components with well-documented roles in the signaling processes occurring throughout the course of disease development and maintenance. Nodes representing cell states were defined based on the hallmark phenotypes of psoriasis to ensure that all the relevant phenotypes can be efficiently captured by the model. The edges in the model represent the causal (activating or inhibiting) interactions between signaling components or transitions between different cell states.

UPMaBoSS is an extension of MaBoSS^14^ that allows simulations of cell population dynamics. In this framework, cells can die, divide, and interact. The size of a cell population is defined by multiplying the probability associated with an active cell node and the overall population size, for the same time point. The overall population size is calculated based on the *Division* and *Death* nodes, reflecting the increase or decrease of cell populations relevant to *t* = 0. In the PsoriaSys model, the keratinocyte is the only cell type that influences the Division and Death nodes, and therefore only the proliferation and death of keratinocytes are captured by the model. Considering that keratinocytes comprise the cell population whose phenotype is most affected by disease progression and alleviation, the population levels of keratinocytes and their physiological states (proliferation versus differentiation) were used as a proxy for the disease course. In addition to proliferation, four more physiological states of the normal and diseased keratinocyte life cycle are represented: the normal, unstimulated keratinocyte (KC node), which ends its life cycle as a fully differentiated keratinocyte (Diff_KC node); the activated keratinocyte that responds to external or internal stimuli and initiates an inflammatory cascade (aKC node); the proliferating keratinocyte (Prol_KC) characteristic of the disease; and lastly, the pre-differentiated keratinocyte (preDiff_KC node), a state of psoriatic keratinocyte in which the cell is unable to differentiate fully. Other stages of the keratinocyte life cycle, such as desquamation, are not included in the model, as keratinocytes in these states are not affected by active immune cells and need not be taken into consideration for this multiscale model.^15^

To ensure that the model can recapitulate known behavior, the sequence of the main events during disease development was confirmed (Figure 1C). As observed from the activation trajectories of the nodes, the model successfully captures the initial responses of KCs to psoriatic triggers that lead to the activation of dendritic cells. Activated dendritic cells then translocate to the lymph node to activate Th naive cells. The time needed for this translocation is encoded as the parameter associated with the activation rate of Th0 cells and captured as a small delay between DC activation and Th activation and differentiation. When in the lymph, secretion of cytokines from DCs shapes the differentiation of Th cells to Th1, Th17, and Th22 subtypes while reducing their ratio to their anti-inflammatory counterpart, Treg cells. Differentiated Th cells then infiltrate the skin and disrupt the KC life cycle by secreting cytokines that promote their proliferation, disrupt their terminal differentiation, and prevent their death. Proliferating KCs secrete cytokines and chemokines that further recruit and promote the survival of the various immune cells. In the meantime, cell types that link innate and adaptive immunity and further enhance inflammatory responses, such as M1 macrophages and neutrophils, are also activated.

### Sensitivity analysis

UPMaBoSS separates the rates of activation and inactivation of each node of the model, providing a means to modulate the timing of events, the behavior, and the long-term activity states of the system. A sensitivity analysis was conducted to evaluate the extent to which these parameters influence the system’s behavior and whether they have any effect on its outcome. The PsoriaSys model contains 155 parameters that determine each node’s activation or inactivation rate. These rates define how quickly an entity will be activated or inactivated during simulations. When available, the parameters were based on observations in the literature. As this type of information is scarce, only 17 parameters could be defined based on prior knowledge (Table S1). All other parameters were set to match generally accepted principles and chosen to be the same for nodes of the same type. For example, cytokine and chemokine ligands were set to undergo rapid degradation/inactivation (i.e., high inactivation rate), resulting from extracellular signal depletion and other mechanisms of inflammation control.^16^ For receptors, the activation rates were updated in each step of the simulations based on the probability of their ligand being active; the more the ligand is produced, the higher the activation rate of its receptor. To test the robustness of the model, a sensitivity analysis was performed by creating versions of the model where each of the parameters is either reduced or increased by 50% of their initial value, and the effects of these parameter changes were observed for the first 200 simulated hours following a psoriatic trigger. This time point was selected based on initial analyses on the model’s behavior where it was observed that the model reaches an asymptotic behavior within this time frame. The deviation of the activation probability in the different model versions from the activation probability in the “wild type” (WT) model was used as a metric of the effect of a parameter on the model’s behavior. In a robust model, these deviations should be small and have a limited effect on the node’s state trajectory.

For most of the nodes, the changes in the model’s parameters do not significantly affect the behavior of the model, with almost all changes in the nodes’ activation probabilities deviating less than 0.1 from the WT probability (Figure S1). While these deviations remained small, the state trajectories of a few nodes are sensitive to changes in certain parameters. The Treg (i.e., Regulatory T cells) node was sensitive to changes in the activation rates of some of its direct regulators; namely the increased activation rate of RAR-related orphan receptor gamma (RORγ) and Aryl hydrocarbon Receptor (AhR) transcription factors and the reduced inactivation rates of Cytotoxic T-Lymphocyte Associated Protein 4 (CTLA4) and IL-12. RORγ and AhR are transcription factors related to T helper cell differentiation toward Th17 and Th22. AhR activity suppresses Treg differentiation,^17^ and Th17 differentiation, mostly driven by RORγ, is mutually exclusive with Treg differentiation.^18^ Furthermore, the activation probability of the dendritic cell nodes (i.e., mDC and pDC nodes) was sensitive to the activation rate of the LL-37/Nucleic acid complex, which is responsible for their activation in response to a psoriatic trigger. Lastly, the probability of active fibroblasts was affected by the reduced activation rate of proliferating keratinocytes affecting the probability of active fibroblasts. The relationship between fibroblasts and proliferating keratinocytes can be traced to the activation of fibroblasts by the KC-derived IL-19, which in turn stimulates keratinocyte proliferation by priming fibroblast to secrete keratinocyte growth factor (KGF).^19^ Comparing the size of the proliferating keratinocyte population between conditions shows that the activation and division of proliferating keratinocytes was not sensitive to changes in any of the model’s parameters, with this population increasing in size at a similar rate as in the “wild type” model. This behavior may result from how the model was built to represent the events of disease progression in the presence of psoriatic stimuli, with an active disease phenotype expected to be reached unless a mitigating perturbation is applied.

### Model validation

Having established that the model can show cell population dynamics consistent with the literature on disease progression (see above), a set of *in silico* perturbations was checked against known observations from a collection of physiological observations under certain conditions, such as disease subtypes or in response to treatment.

**Figure 2:**
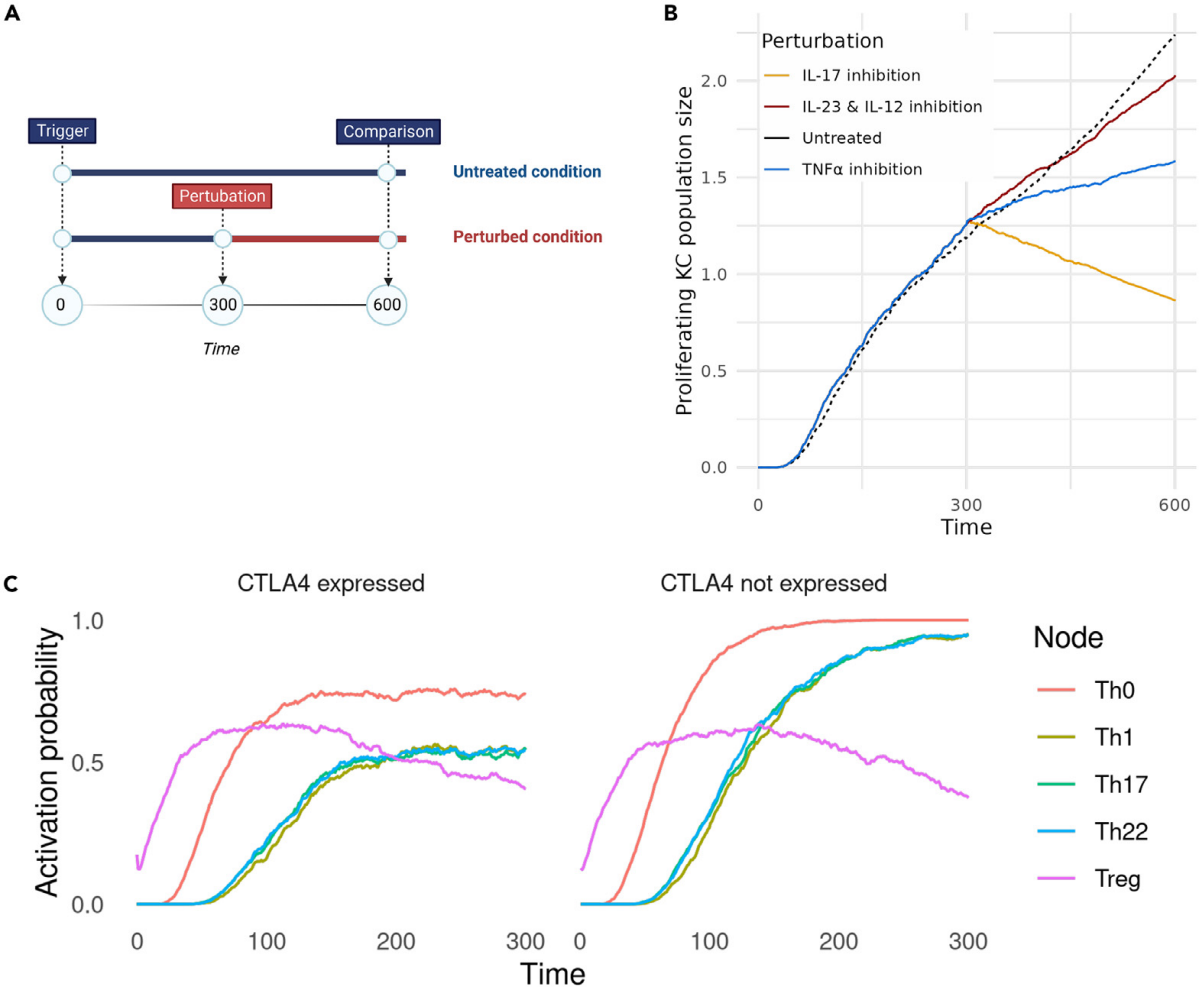
Perturbation analysis of the PsoriaSys model (A) Diagram illustrating simulated perturbation scenarios. In the untreated conditions, the model’s behavior was simulated from an initial psoriatic trigger at time 0 to time 600. In the perturbed conditions, the model’s behavior mirrored the untreated conditions up to time 300, where a perturbation was introduced. To assess the impact of the perturbation on the model’s behavior, the activation probabilities at time 600 were compared between the untreated and perturbed conditions. (B) The effect of biologics in psoriasis on the population size of proliferating keratinocytes compared to the population size in untreated conditions (dashed black line). The different perturbations are denoted by colors (IL-17 inhibition in yellow, TNFα in blue, and IL-23/IL-12 in dark red). The x axis represents time and the y axis represents the relative size population of proliferating keratinocytes. (C) Comparison of T-helper activation when T-helper cells express CTLA4 (left panel) and T-helper activation when CTLA4 is not expressed (right panel). The x axis represents time, and the y axis represents the probability of activation of each node.

The effects of the most frequent treatment options were also validated by testing the response of the system to the inhibition of IL-17, TNFα, IL-12, and IL-23, all of which are the main targets of the biologics used to treat moderate-to-severe psoriasis.^8^ The effect of treatment was simulated by using ‘chained’ simulations (Figure 2A). First, a trigger is applied to the system, encoded as the activation of the “*Trigger”* node. Upon this trigger, the system reaches an active psoriatic state. After this psoriatic state is reached, a second simulation is performed, in which an *in silico* perturbation is applied to the psoriatic system state that resulted from the initial trigger. In the case of inhibitory perturbations, the state of the perturbation target was fixed at 0, while in activating perturbations the target’s state was fixed at 1. The treatment effect is then compared to the predicted trajectory of the individual cell types where no treatment is applied.

Current biologic treatments mostly target the IL-23/IL-17 pathogenic axis in different stages of the disease development. IL-17 is a multifunctional cytokine widely characterized as a key driver of psoriasis by regulating cell fate proliferation and differentiation and sustaining the release of inflammation-promoting agents, such as antimicrobial peptides.^3^^,^^20^ Biologics targeting IL-17, for instance ixekizumab and brodalumab, have shown excellent efficacy in treating moderate-to-severe psoriasis and are relatively well-tolerated with long-term maintenance of treatment responses.^21^ Targeting IL-17 in the multiPSO model leads to the biggest reduction of proliferating KC population (∼64% decrease) compared to the simulated single-target treatments (Figure 2B; Table S3). However, IL-17 inhibition has a relatively limited effect on the rest of the cell types (Table S3). As keratinocytes and generally non-hematopoietic cells are the main targets of IL-17,^20^^,^^22^ it was hypothesized that by inhibiting IL-17, the normal KC life cycle is restored, which also leads to the limited production of inflammation-inducing agents and the subsequent reduction of other cell types. Therefore, the inhibition of other immune cells could be presented as a secondary effect in the model.

TNFα is involved in the earlier events of psoriasis and drives the induction of dendritic cells and their maturation.^23^ Additionally, TNFα acts synergistically with IL-17 on KC responses, enhancing the expression of main inflammatory drivers, KC-derived cytokines, and other immunomodulating molecules.^24^ Several TNF inhibitors, such as adalimumab and etanercept, have been approved for treating moderate-to-severe psoriasis; however, all with a variable efficiency among different patients.^25^ The inhibition of TNFα can induce various responses in patients, including the induction of paradoxical psoriasis, a type of psoriasis that is Th cell-independent and driven by Type-I IFN signaling.^23^^,^^26^ The inhibition of TNFα in the PsoriaSys model results in a 15% reduction in the KC population (Figure 2B; Table S3). In addition to its effect on KC, TNFα amplifies inflammatory responses in several ways. Fibroblasts stimulated with TNFα and IL-8 are not able to produce IL-10, a potent anti-inflammatory cytokine.^27^ Additionally, IL-10 production in response to TNF blockade has been observed *in vitro.*^28^ With the reduction of proliferating KC and subsequently the KC-derived IL-8, fibroblasts secrete IL-10 activating anti-inflammatory M2 macrophages. The production of IL10 leads to the eventual repression of Th22 cells and, therefore, lower IL-22 levels. According to the model, TNF blockade reduces the KC population by inducing their death. *In vitro* experiments on human epidermal keratinocytes (HEKs) showed that IL-22 plays a strong antiapoptotic role in the cell fate of KC, independently of IL-17.^29^ This mechanism could explain the reduced population of proliferating keratinocytes.

Lastly, the effect of inhibiting IL-12 and IL-23 was tested. Both cytokines promote T-cell-mediated responses by shaping the differentiation of Th cells toward the Th1 and Th17 phenotypes. Ustekinumab is an antibody that blocks IL-12 and IL-23 signaling by binding and neutralizing the shared p40 subunits of the two cytokines.^30^ IL-12/IL-23 inhibition reduces the KC population by 34% (Figure 2B; Table S3) and completely inhibits Th cell activation and differentiation. Comparative studies between biologics targeting IL-17, IL-23, TNF, and IL-12/IL-23 showed that IL-17 and IL-23 inhibitors have a higher efficiency than IL-12/IL-23 and TNF inhibitors.^25^ The model successfully predicts a higher effect of IL-17 inhibition compared to TNF inhibitors, but IL-23 and the combinatorial inhibition of IL-12 and IL-23 show similar levels of effect on KC populations. While the model can be used to compare the probabilities of a cell being activated or inhibited between different conditions, other more quantitative models, such as pharmacodynamics/pharmacokinetics models,^31^ could be used to quantify treatment effects in more detail.

In addition to treatment responses, we also found that the model could distinguish between disease development of mild and more severe cases of psoriasis. In the study of,^32^ CTLA4 was proposed to be upregulated in patients with milder psoriasis. T cell activation is regulated by co-stimulatory molecules that can either promote or inhibit their activation. CD28 is a receptor in T naive cells that binds to CD80 and CD86 ligands on the surface of antigen-presenting cells (such as DCs). CTLA4 is another co-stimulatory molecule on the surface of T cells similar to CD28, which competitively binds the CD80/CD86 ligands with a much higher affinity than CD28. Contrary to CD80, CTLA4 elicits an inhibitory signal for T cell activation. After the activation of T cells by CD80/86-CD28 interactions, CD28 is internalized and degraded, and CTLA4 translocates to the cell surface. This negative regulation provides a control mechanism to regulate T cell activation and inflammation, which does not occur in psoriasis. The effect of CTLA4 was encoded in the regulation of the Th0 node by increasing the rate of its downregulation when CTLA4 is expressed. The development of the disease in two model instances, one with a functioning CTLA4 and one without, shows that CTLA4 expression can prevent full activation of Th cells, potentially leading to a milder manifestation of the disease, as shown in Figure 2C.

### Perturbation analysis

Having confirmed that the model adequately represents the basic regulatory network underlying psoriatic development, a systematic perturbation analysis was performed to identify new potential drug intervention points. While targeting each druggable component, the inhibition of inflammatory components and the activation of anti-inflammatory components were tested. The effect of each perturbation was assessed by model readouts that mirror measurements commonly used in a clinical setting to assess treatment response, namely reduction of skin thickening (represented as the proliferating KC population size in the model), T-helper and Treg activation, and immune cell recruitment. For each of the treatments, the response was quantified as the change in probability of a node to be activated by the formula:

$$\left(Prob_{treated}\;-\;Prob_{WT}\right)\;/\;Prob_{WT}\;*100$$

The responses to the different treatments were highly variable. The results of all tested perturbations can be found in Table S3. In addition to the effectiveness of the known biologics (Figure 2B), the inhibition of Tyrosine-Kinase 2 (TYK2) was among the most effective perturbations concerning the aforementioned criteria, as it reduces KC proliferation (by 33%) and abolishes the activation of Th cells. TYK2 is part of the JAK-STAT signaling pathway that mediates the expression of several drivers of psoriasis, such as Type I IFNs, IL-12, and IL-23, making TYK2 an integral part of inflammation and immune response regulation. A similar effect with the TYK2 inhibition is predicted for other components of the IL-23/IL-17 axis, such as the inhibition of IL-23 and STAT3. However, the differentiation of the Th1 cells is not inhibited, as IL-12 expression, the driver of Th1 differentiation, is not affected in any way by targeting the IL-23/IL-17 axis.

Another promising target proposed by the model is prostaglandin E2 (PGE2), whose inhibition reduces the size of the proliferating KC population and inhibits Th cell activation. PGE2 is a lipid mediator produced from arachidonic acid (AA) and elicits a wide range of biological effects in inflammation and its resolution. PGE2 acts through four main Prostaglandin E2 receptors (EP1-EP4), all of which are connected to a diverse set of downstream pathways. EP2 and EP4 are upregulated in psoriasis^33^^,^^34^ and are the primary receptors through which PGE2 exerts its pathogenic effect in psoriasis. Due to their partial cross-talk, the inhibition of either EP2 or EP4 does not significantly reduce KC proliferation. However, EP4 inhibition shows a mild effect on the reduction of early activated immune cells (e.g., inflammatory DCs), naive Th cell activation, and, ultimately, the complete inhibition of Th17. An activating perturbation of IL-10 was among the few tested perturbations which, in addition to Th cell inhibition, leads to the activation of Treg cells. Additionally, it results in a phenotype shift of macrophages from the pro-inflammatory M1 phenotype to the anti-inflammatory M2 phenotype, and a reduction of neutrophil activation. However, it shows no effect on the KC populations. This observation corroborates the results of clinical trials on IL-10 therapeutics, where moderate improvement of psoriasis severity was attributed to the modulation of immune cell activation, with no observed direct effect on keratinocytes.^35^

### Identification of high-influence node sets for combinatorial perturbation

After identifying effective single-node perturbations, the model was used to predict the effectiveness of combinatorial perturbations. As psoriasis is characterized by multiple feedback loops that amplify and sustain the disease’s phenotypes,^4^^,^^36^ it was hypothesized that the targeting of entities that are part of those feedback loops might restore normal phenotypes. A Feedback Vertex Set (FVS) is a set of nodes that includes at least one node from each feedback loop of a model. In the PsoriaSys model, 27 nodes (∼34% of the total nodes) are part of at least one feedback loop. However, due to the size of the FVS, targeting all of its components would not be a feasible option in experimental or clinical settings. To overcome this challenge, network propagation metrics were used to identify subsets of the complete FVS (also called minimum FVS) that could potentially serve as drug targets against psoriasis. Minimum FVS of two nodes were selected, based on the fact that pairwise combinations are the most commonly investigated in clinical settings. These subsets were ranked based on the overlap of the propagation metrics as described in.^37^ A higher ranking of a node pair indicates a greater expected influence on the model’s states when the pair is perturbed.

In total, 333 two-node FVS were identified, and 62 of those pairs included non-druggable nodes. Druggable nodes represent transcription factors, ligands, or receptors, in contrast to nodes representing cell types (e.g., Th0) or cell states (e.g., proliferating keratinocytes). Among the 217 druggable target pairs, cytokines, which are considered drivers of the disease and are already targets of the standard therapy options, were dominating. The top 20 target pairs (Table S2) were further analyzed. If both a cytokine and its receptor appeared with the same second target, only the cytokine inhibition was simulated as single-node perturbation analysis showed that inhibiting either a cytokine or its receptor had almost identical effects on the system.

**Figure 3:**
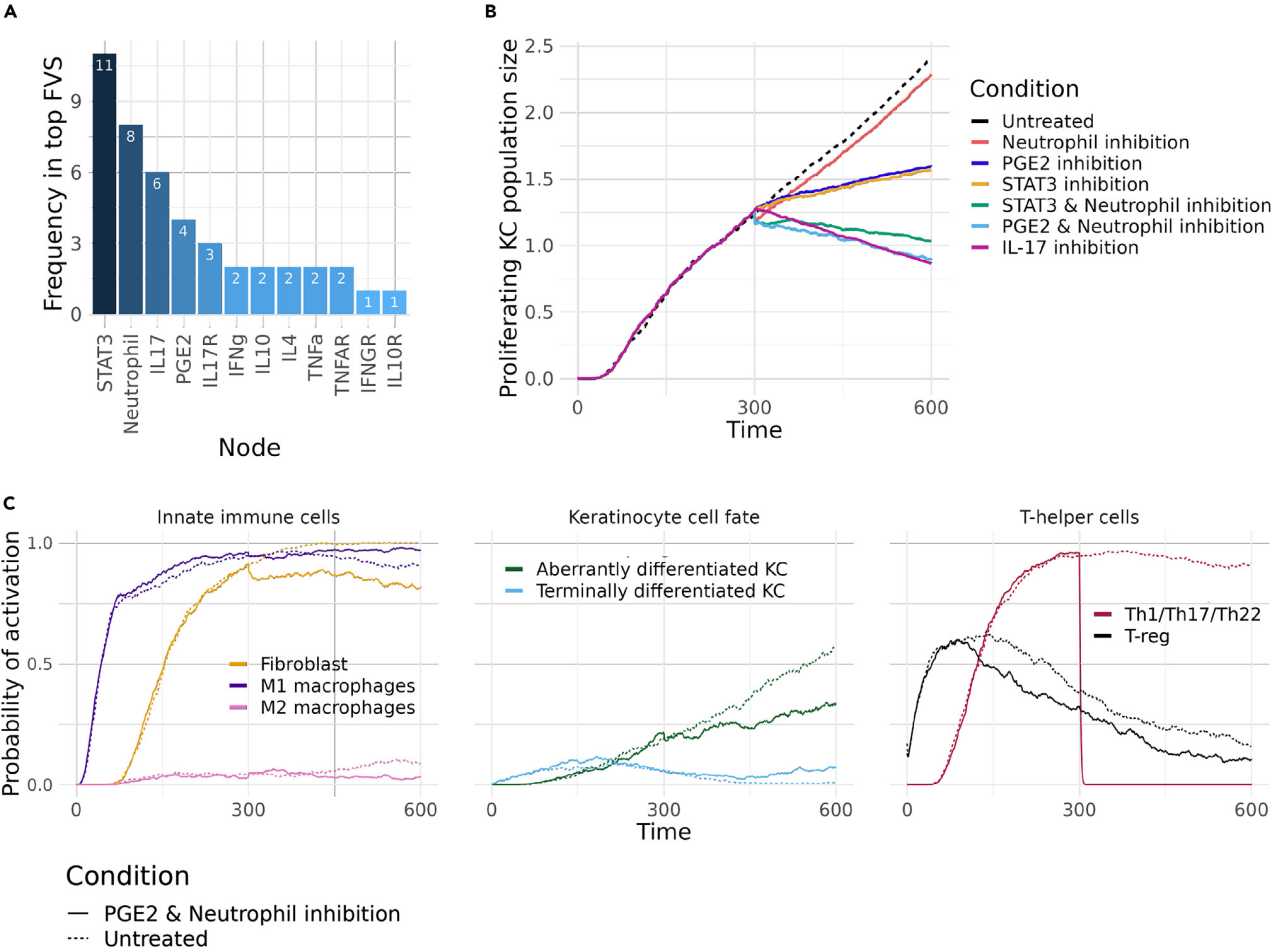
Identification of combinatorial perturbations using network metrics (A) Frequency in which a node was included in minimum Feedback Vertex Sets (FVS), as identified by the intersection of three network propagation metrics (i.e., PRINCE, modifiedPRINCE, and CheiRank). (B) Comparison of the population size of proliferating keratinocytes (x axis) for the combinatorial targeting of neutrophils and PGE2 production, or neutrophils and STAT3. The population sizes for untreated conditions (dashed line) and the inhibition of neutrophils, PGE2, and STAT3 alone are included for reference. (C) Trajectories of the probability of activation of the cell nodes that respond to the combinatorial inhibition of neutrophils and PGE2 compared to their trajectories in untreated conditions (dashed lines). The y axis shows the probability of activations and the x axis shows Time.

Interestingly, the only cell nodes in the FVS were those representing proliferating keratinocytes, Th1, Th17, and neutrophils. The role of KC and Th cells in the self-sustained inflammatory cycle of psoriasis is well-described, therefore, suggesting that their reduction would reverse psoriatic phenotypes. While neutrophil recruitment is a histological characteristic of psoriasis, its pathogenic role in the disease remains unclear. To explore the potential of neutrophils as a target we tested the effect of their inhibition with the PsoriaSys model. Neutrophils are one of the primary innate immune cells and are activated by a diverse set of inflammatory stimuli. Neutrophils can link innate and adaptive immunity by interacting with DCs and T cells and are attracted to psoriatic lesions by several chemokines released by KCs and T cells. On site, they induce respiratory burst, degranulation, and neutrophil extracellular traps (NETs) formation. Such processes can stimulate pDCs by activating TLR receptors, which start the inflammatory cascade that leads to the pathogenesis of psoriasis. Additionally, neutrophils can amplify immune responses through several mechanisms, such as the release of proteinase-3 that cleaves pro-IL-36 to its activated form and the release of IL17, for which it is one of the main sources.^38^ Among the top 20 target pairs proposed (Table S2), neutrophils were implicated in 8 of them (Figure 3A). Notably, the combinatorial targeting of components of the IL-23/IL-17 axis along with neutrophil activation was ranked as the most influential target pair. In the model, the role of neutrophils is restricted to their effect in psoriatic signaling and cytokine release, while processes such as the formation of NETs are difficult to represent in the current modeling framework. The inhibition of the neutrophils alone has only a limited effect on the psoriatic phenotype, reducing proliferating keratinocytes by only 5% and not affecting the main immune cells. However, a potential synergistic response is observed when neutrophil inhibition is combined with the inhibition of PGE2 or STAT3. The single inhibition of PGE2 or STAT3 reduces proliferating keratinocytes by ∼33%. When combining PGE2 and neutrophil inhibition, the population of proliferating keratinocytes is reduced to levels (63%) similar to the most effective single inhibition (i.e., IL-17 inhibition). Additionally, this combination restores the terminal differentiation of keratinocytes, as captured by their increased activation probability, and it reduces the probability of activation of the pre-differentiated populations. Furthermore, it completely abolishes the activation of all Th cell populations, ranking this combination among the most effective for restoring a normal phenotype from a psoriatic state. When the inhibition of neutrophils is combined with the inhibition of STAT3, we also notice a significant reduction of proliferating KCs (57%) (Figure 3B). For the other cell types, the changes are mostly STAT3-driven and only a limited effect on Th1 activation or innate immune cells is observed.

Next on the ranked list of target pairs, the inhibition of IL17R and STAT3 was simulated. The combination of IL-17 and STAT3 leads to a similar reduction of proliferating keratinocytes as the inhibition of IL17 alone. However, it also reduces other immune cells, including M1 macrophages, fibroblasts, and all Th cells, and increases the activation of the anti-inflammatory Treg cells by 23%. STAT3 has a multifaceted role in psoriasis and influences several cell types. Activated by IL-23 in Th cells, STAT3 induces the expression of RORγ, which promotes Th17 responses and the expression of IL-17A, IL-17F, IL-22, and IL23R, further amplifying inflammatory responses. In keratinocytes, STAT3 is activated downstream of IL-17 and IL-22 responses^19^^,^^39^ and produces CCL20 in an IL-1b-induced way.^40^ Therefore, this double inhibition could inhibit psoriatic phenotypes by inhibiting the alternative ways the uncontrolled inflammation might arise. The results of other combinations with IL-17, namely neutrophil inhibition or IL-4 induction, did not differ from IL-17 single inhibition results. For the remaining target pairs, the combinatorial perturbation of the IL-23/IL-17 axis and entities with a role in inflammation amplification were also highly ranked. These combinations include the double inhibition of STAT3 with either PGE2, TNFα, or IL-4 (Figures 3B and 3C). However, these combinations show limited, if any, additional effects compared with the single perturbations. Similarly, none of the combinations that included IL-4, resulted in an effective reduction of psoriatic phenotypes. IL-4 is the only cytokine that shifts the differentiation of Th cells toward a Th2 phenotype.^41^ The imbalance between Th1 and Th2 cytokines is associated with the development of psoriasis^42^ and correlated with disease severity.^43^ Shifting the T cell differentiation toward Th2 by administering IL-4 has been shown to alleviate symptoms in patients with severe psoriasis.^41^ However, the model could not replicate this observation, potentially suggesting some gaps in the knowledge of IL-4-driven mechanisms and therefore their underrepresentation in the model.

## Discussion

We have developed a logical, multiscale model of psoriasis (PsoriaSys model) that integrates molecular interactions, signal transduction, and cell-to-cell communication between psoriatic cells and their surrounding environment. The PsoriaSys model represents the biological events that occur upon the presence of psoriatic triggers that lead to the initiation of the disease and mechanisms and pathways driving disease development and maintenance. The model links the dysregulated signal transduction processes to the secretion of growth factors, cytokines and chemokines, and it includes intercellular communication between psoriatic skin cells, namely keratinocytes and immune cells.

This PsoriaSys model uses logical formalism combined with stochastic simulations. Logical formalism allows the discrete modeling of psoriatic interactions. In contrast, the stochastic simulations allow the exploration of temporal evolution beyond the strict state changes supported by this qualitative framework and the analysis of the probabilities of nodes to reach a specific state. Therefore, the model is able to capture the reduction of psoriatic phenotypes, even when not fully inhibited, allowing a better description of the effect of perturbations. This combination was valuable for replicating the development of psoriatic phenotypes in response to psoriatic triggers and emulating the known effect of drug perturbations. Additionally, model instances, where the parameters of the nodes were adjusted to represent specific biological scenarios, allow us to capture the effects of some of the known sources of patient heterogeneity. This was showcased by simulating the effect of lack of CTLA4 expression, where the model recapitulates the effect of CTLA4 in immune cell activation. CTLA4 is a co-inhibitory molecule that acts by limiting T helper cell hyperactivation, and CTLA4 expression has been found to be inversely correlated with psoriasis severity.^32^^,^^44^ Having validated the descriptive properties of the model, it was next used to predict the effect of perturbations of single entities or their combinations. It was shown that the model correctly identifies IL-17 inhibition as the most effective in reducing the population size of proliferating keratinocytes. In clinical settings, IL-17 blockade through biologics has provided a treatment option against moderate-to-severe and severe cases^21^ with unprecedented efficacy. Among other effective perturbations, the inhibition of PGE2 or TYK2, and the activation of IL-10 were proposed. Recently, the first TYK2 inhibitor (i.e., deucravacitinib) was approved by the US Food and Drug Administration (FDA) for the treatment of moderate-to-severe plaque psoriasis. The model’s ability to predict known targets highlights that the model accurately represents the biological regulatory wiring that drives the pathogenesis and development of psoriasis. The role of PGE2 is multifaceted in psoriasis, with PGE2 being involved in multiple aspects of disease development. Several studies show that PGE2 shifts adaptive immunity toward Th1 and Th17 responses by affecting DCs,^45^ priming the expansion of Th17 cells, and promoting the differentiation of Th1 cells.^46^ The effect of PGE2 on cell fate decisions of KC has been previously described by our work on the computational and experimental modeling of KC behavior upon cytokine and PGE2 stimulation.^33^ Inhibitors against cPLA2a, the phospholipase regulating PGE2 production, have shown high efficacy against plaque psoriasis in clinical trials.^47^ While several well-established psoriasis drugs are reducing EP and, subsequently, PGE2 levels, the direct inhibition of EP receptors has not been fully explored in psoriasis, although it is being investigated in other diseases, such as treatment of solid tumors.^48^

Furthermore, using network propagation metrics, we identified pairs of model components whose combinatorial targeting was more likely to control the system’s behavior. The inhibition of neutrophil action together with PGE2 or STAT3 reduced psoriatic phenotypes to similar levels as the IL-17 blockade. Neutrophils act as immune regulators in psoriasis, bridging innate and adaptive responses,^38^ and their accumulation in psoriatic skin is considered a hallmark of psoriasis.^49^ During degranulation and respiratory burst, neutrophils release, among others, granule-derived serine proteinases, such as NE, proteinase 3, and cathepsin G, which can activate by cleavage various psoriatic cytokines, such as TNFα and IL-36.^38^ Furthermore, neutrophil clearance is associated with successful treatments, for instance, anti-IL-17 agents.^38^^,^^49^^,^^50^ In general, anti-IL-17 drugs are hypothesized to act on neutrophil-derived IL-17 and disrupt neutrophil and keratinocyte crosstalk.^22^ Based on the model, we suggest that neutrophils could serve as a promising target for treating psoriasis, either alone or in combination with other components of the IL-17/PGE2 axis components.

### Limitations of the study

As in every modeling effort, the model’s ability to represent specific events is limited by the available knowledge about the mechanisms underlying such events. Additionally, technologies such as single-cell RNA sequencing could aid with defining and fine-tuning specific mechanisms and parameters of the model, such as the cell population sizes in normal and treated conditions. However, only a few datasets are currently available for psoriasis. Additional bias could be attributed to decisions taken during the modeling process. For instance, the model represents the proliferation only of keratinocytes. While the increase of immune cell populations due to their proliferation and recruitment is a characteristic of psoriasis, immune cell proliferation is not explicitly represented in the model. As the activation of T-helper cells is unrelated to their effector functions and cytokine secretion^51^ we did not consider this necessary for the computational analysis of psoriasis development. Therefore, the population levels of keratinocytes and their physiological state were used as a proxy for the progress of the disease, considering that keratinocytes are the primary responders to disease progression or alleviation. Lastly, certain mechanisms that require a spatial description, such as cytokine diffusion or NETosis, cannot be explored in detail with the UPMaBoSS framework. To account for this, the model could be expanded to include such spatial properties and to better represent the spatial organization of the skin layers. The expanded model could be then analyzed as an agent-based model with the PhysiBoSS framework.^52^ With the current framework, we have demonstrated the potential of multiscale models of diseases to investigate the underlying disease pathogenesis and to identify potential targets for improving the efficacy of targeted therapies. In the future, the PsoriaSys model could be expanded to include other pertinent cell types and signaling pathways. An interesting and promising aspect, which could be explored through a dynamic Boolean model, involves the understanding of the development of tissue-resident memory cells, a phenomenon associated with disease recurrence.^53^ Moreover, enhancing the model with biomarkers and patient-specific data holds promise for predicting alternative paths of disease advancement, thereby offering insights into variations of treatment responses.

## STAR★Methods

### Key resources table

**Table:** 

REAGENT or RESOURCE	SOURCE	IDENTIFIER
**Deposited data**		

Logical model of psoriatic system - PsoriaSys model	This paper	https://github.com/Eirinits/PsoriaSys_model and https://www.ebi.ac.uk/biomodels/MODEL2308300001 and https://doi.org/10.5281/zenodo.10351225

**Software and algorithms**		

bioLQM	http://colomoto.org/biolqm/	https://doi.org/10.3389/fphys.2018.01605
GINsim	http://www.colomoto.org/software/ginsim.html	https://doi.org/10.1016/j.biosystems.2009.04.008
MaBoSS	http://www.colomoto.org/software/maboss.html	https://doi.org/10.1186/1752-0509-6-116
UPMaBoSS	https://maboss.curie.fr/	https://doi.org/10.3389/fmolb.2022.800152
PsoriaSys model analysis code	This paper	https://github.com/Eirinits/PsoriaSys_model and https://doi.org/10.5281/zenodo.10351225
Feedback Vertex Set (FVS) identification	Newby et al.^37^	https://doi.org/10.1063/5.0080843

### Resource availability

#### Lead contact

Further information and requests for resources should be directed to and will be fulfilled by the lead contact, Eirini Tsirvouli (eirini.tsirvouli@ntnu.no)

#### Materials availability

This study did not generate new unique reagent.

#### Data and code availability

• No biological data were produced in this study.
• All original code for the analysis of the model has been deposited at Zenodo and as a GitHub repository and is publicly available as of the date of publication. The model files have also been deposited at the BioModels repository. DOIs are listed in the key resources table.
• Any additional information required to reanalyze the data reported in this paper is available from the lead contact upon request.

### Method details

#### Model construction

A Prior Knowledge Network (PKN) encompassing the main inter- and intra-cellular signaling events taking place in the development of psoriasis was manually curated from scientific publications about the disease and its progression. The PKN consists of biological entities (nodes) with a reported role in the disease and their regulatory interactions (edges). Each node represents a cell type in various physiological states (called Cell nodes), a ligand or its respective receptors, a transcription factor, or other intermediate signaling proteins (collectively called Signaling nodes). The interactions between nodes were collected from the available literature and databases with causal molecular interactions, such as SIGNOR 3.0 (Perfetto et al., 2016).^54^ Over decades of research on the disease, multiple innate and adaptive immune cells have been described for their role in psoriasis. However, the model focuses on the disease's main cellular players, whose role is already well established.

Once the PKN was constructed, it was converted to a Boolean model. In Boolean models, the activity or state of a node can only take two possible values, 0 or 1. Nodes with a state of 0 are considered inactive, while active nodes take a value of 1. The state of a node is defined by its logical rules that define how the state of a node changes depending on the activity and the combinations of the nodes that regulate it. The logical rules were defined manually using the logical formalism AND, OR, and NOT. Effector molecules, such as cytokines, were defined as active when both the cell type producing them and their respective regulator (e.g., transcription factor) were active. For example, the AMP node representing antimicrobial peptides was considered active when both Prol_KC and STAT3 were active (AMP = Prol_KC AND STAT3). Receptor nodes were activated by their respective ligands. For instance, the IL-10 receptor is activated by IL-10 (IL10R = IL10). Nodes representing differentiated Th cells were activated by their master regulators. To illustrate, the differentiation of Th cells from naive (Th0) to Th-17 cells was denoted as follows: Th17 is activated if both Th0 is active AND RORγ is active (Th17 = Th0 AND RORgamma). This scheme was followed unless information from the literature suggested otherwise.

#### UPMaBoSS model

MaBoSS is based on Markov Chain processes applied to a Boolean network. A MaBoSS simulation computes trajectories of probabilities for nodes to be active over time. UPMaBoSS extends on the MaBoSS framework by adding dynamical properties such as the evolution of cell population size over time: cells can die, divide and communicate. The population update is done synchronously throughout the simulation (at every *t* step) until the maximum time is reached. The stochastic Boolean simulation framework UPMaBoSS^13^ was used to approximate continuous behaviors, calculate the probabilities of node or phenotype activation, and estimate the cell population sizes.

First, to allow the simulation of population dynamics, two nodes representing ‘Division’ and ‘Death’ were added to the PsoriaSys model to allow the population dynamics simulation. The probabilities of activation are doubled for trajectories with the Division node equal to 1, and for trajectories with the Death node equal to 1, the probabilities are set to 0. Each node of the model was assigned rates of activation and inactivation. When available, the parameters were defined based on available literature reporting on specifically psoriatic conditions or any other skin inflammatory states. As this type of measurement is scarce, only 17 parameters were defined based on prior knowledge (Table S1). The remaining parameters were defined to be the same for nodes of the same type. For example, cytokine and chemokine ligands were set to undergo rapid degradation/inactivation (i.e., high inactivation rate), resulting from extracellular signal depletion and other mechanisms of inflammation control.^16^ For receptors, the activation rates were updated in each step of the simulations based on the probability of their ligand being active; the more of a ligand produced, the higher the activation rate of its receptor. Due to the high number of parameters, a sensitivity analysis was performed to assess the robustness of the model and ensure that the assigned parameters were biologically reasonable. Each parameter was either reduced or increased by 50% of its initial value, similar to the sensitivity analysis performed in Checcoli et al.^12^

#### Minimum feedback vertex set (FVS) identification

The presence of feedback loops (or circuits) in a network has been shown to play a role in its dynamics: its ability to reach either multiple stable states in the presence of positive feedback loops or cyclic attractors in the presence of negative feedback loops.^55^ The control of nodes that are part of such feedback loops (collectively called FVS) can be used to drive the network towards a target stable state. As a network can include numerous nodes that are part of the network’s FVS, a minimal set of nodes that can drive the system toward a desired state can be identified (minimum FVS). As this set of nodes is not unique, the intersection of three network propagation measures was used to rank 2-node combinations based on their ability to drive the model toward a desired state and, by extension, identify intervention points (i.e., drug targets) that would lead to psoriasis resolution. These topological measures included PRINCE propagation, Modified PRINCE, and CheiRank random walk, and their intersection is the highest performing for identifying the most influential minimum FVS.^37^ The analysis was performed with a predefined minimum FVS size of two so that the identified perturbations would reflect feasible interventions to treat psoriasis. The analysis requires Python 3.8. Instructions on creating a Conda virtual environment are included in the respective notebook.

## Supplemental information

Document S1. Figure S1 and Tables S1 and S2

Table S3. Node reduction in all tested perturbations, related to Figure 2 and the “Perturbation analysis” section of Results)
