# Supplementary material for: Dynamic Boolean modeling of molecular and cellular interactions in psoriasis predicts drug target candidates
Source: iScience. 2024 Jan 11;27(2):108859. doi: 10.1016/j.isci.2024.108859 (PMC10831929; doi:10.1016/j.isci.2024.108859)

iScience, Volume *27*

Supplemental information

Dynamic Boolean modeling of molecular and cellular interactions in psoriasis predicts drug target candidates

Eirini Tsirvouli, Vincent Noël, Åsmund Flobak, Laurence Calzone, and Martin Kuiper

Table S1. [Rates and parameters curated by scientific literature], related to Figure 1.

Event	Paper	Time	How much?
DC migration to lymph	https://www.nature.com/articles/nature02238	24 h	
DC-T cell contact	https://www.nature.com/articles/nature02238	30 min	
T-cell proliferation	https://www.nature.com/articles/nature02238	24 h	
Neutrophil infiltration	https://www.ncbi.nlm.nih.gov/labs/pmc/articles/PMC2617712/	24 h	
KC turnover (normal)	https://www.karger.com/Article/Fulltext/495291	50 days	
psoKC turnover	https://www.karger.com/Article/Fulltext/495291	5 days	
KC cell cycle	https://www.sciencedirect.com/science/article/pii/0923181194900574?via%3Dihub	13 days	
normal epidermal apoptosis index	https://royalsocietypublishing.org/doi/10.1098/rsif.2014.1071		0.12%
psoriatic epidermal apoptosis index	https://royalsocietypublishing.org/doi/10.1098/rsif.2014.1071		0.04%

Complete life cycle of KC	https://mmegias.webs.uvigo.es/02-english/8-tipos-celulares/queratinocito.php	1 month	
fold change of psoriatic SC proliferation	https://royalsocietypublishing.org/doi/10.1098/rsif.2014.1071		4

Table S2. [Ranking of 2-node perturbations. The propagation consensus score is the score given by the consensus of three network propagation measures: PRINCE, modified PRINCE, and CheiRank.], related to Figure 3

Propagation consensus score	Target 1	Target 2	Propagation consensus score	Target 1	Target 2
97	STAT3	Neutrophil	87	TNFα	Neutrophil
95	IL17	Neutrophil	86	IFNg	Neutrophil
95	IL17R	STAT3	86	STAT3	IL4
94	STAT3	PGE2	86	IL17	PGE2
93	IL17R	Neutrophil	85	IL17R	PGE2
93	IL17	STAT3	84	STAT3	IL10
92	PGE2	Neutrophil	84	STAT3	IFNg
90	TNFAR	STAT3	84	STAT3	IL10R
89	STAT3	TNFα	83	TNFAR	Neutrophil
88	IL4	Neutrophil	82	STAT3	IFNGR

Figure S1. [Sensitivity analysis results. Difference between the activation probability of each cell node between the wild-type model and model instances with adjusted activation/inactivation rates.], related to the “Sensitivity analysis” section.

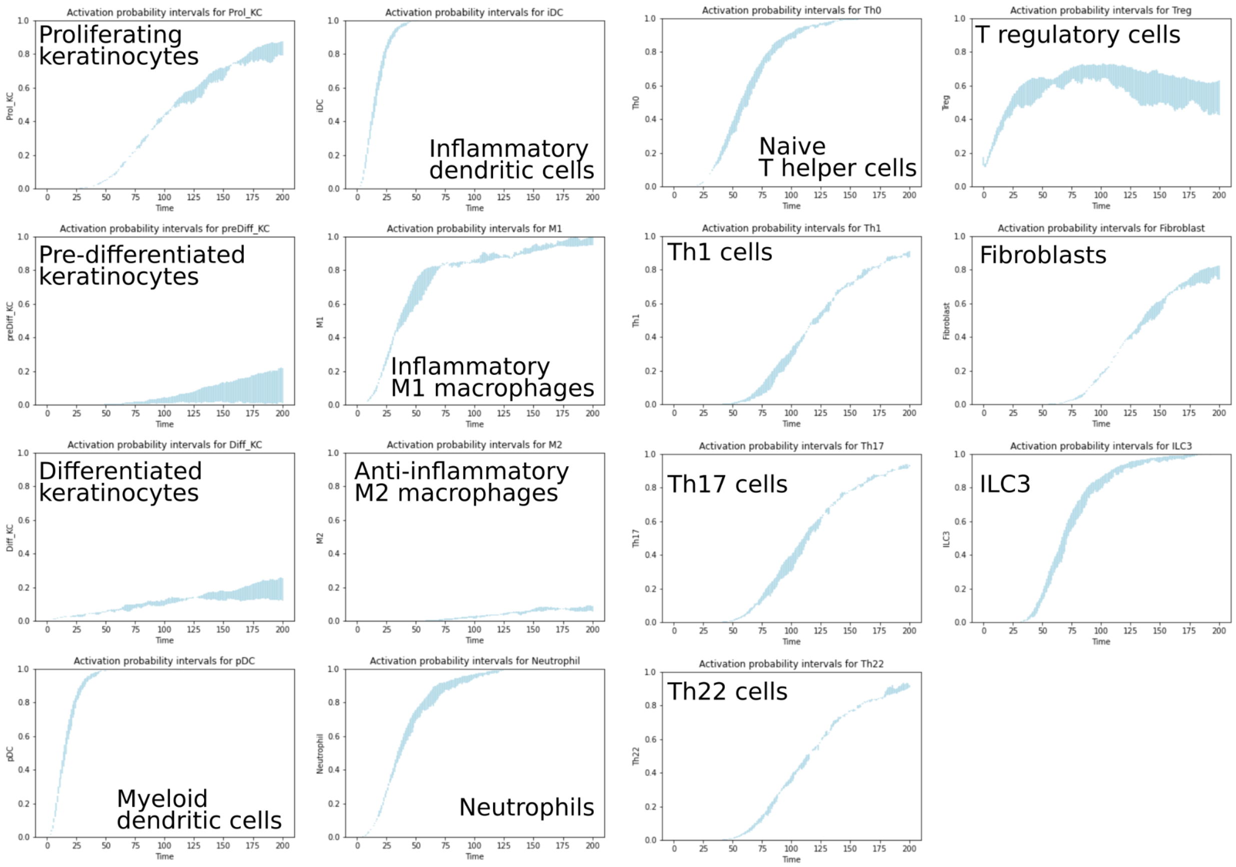

Supplement: Document S1. Figure S1 and Tables S1 and S2 [file mmc1.pdf]
